# Conventional and Emerging Biomarkers for Predicting Post-Ablation Atrial Fibrillation Recurrence: An Updated Systematic Review

**DOI:** 10.14740/cr2231

**Published:** 2026-08-25

**Authors:** Naveed Mohsin, Martin Eugene Matsumura, Tariq Ahmad, Kaif Ul Sahar, Lubna Mohammed

**Affiliations:** a Department of Hospital Medicine, Geisinger Wyoming Valley Hospital, Wilkes-Barre, PA 18711, USA; b Department of Cardiology, Geisinger Wyoming Valley Hospital, Wilkes-Barre, PA 18711, USA; c Department of Sciences, University of Kashmir, India; d Department of Research, California Institute of Behavioral Neurosciences & Psychology, Fairfield, CA 94534, USA

**Keywords:** Biomarkers, Atrial fibrillation recurrence, Catheter ablation

## Abstract

**Background:**

Atrial fibrillation (AF) recurrence after catheter ablation remains common and difficult to predict using conventional clinical risk models. Circulating biomarkers reflecting inflammation, myocardial remodeling, metabolic dysfunction, and neurohormonal activation may improve risk stratification. This systematic review provides an updated synthesis of conventional and emerging biomarkers associated with post-ablation AF recurrence.

**Methods:**

This systematic review was conducted in accordance with PRISMA 2020 guidelines. A comprehensive search of databases (PubMed/Medline, Cochrane Library, ScienceDirect, Europe PubMed Central, and Google Scholar) was performed for studies published between January 2020 and March 2025. Eligible studies included observational cohorts, systematic reviews and meta-analyses evaluating biomarkers associated with AF recurrence after catheter ablation with ≥ 12 months follow-up. Eligible studies were critically appraised using appropriate quality assessment tools. Due to heterogeneity in study design, biomarker measurement, and effect reporting, a qualitative synthesis was performed.

**Results:**

A total of 27 studies encompassing 26,702 participants and 30 biomarkers were included. AF recurrence rates ranged from 15.7% to 51% over follow-up periods of 12–36 months. Stronger predictors identified in larger cohorts included bone morphogenetic protein-10 (BMP-10), homocysteine, triglyceride glucose index, and plasma carbohydrate antigen-125. Among natriuretic peptides, N-terminal pro-B-type natriuretic peptide and mid-regional pro-atrial natriuretic peptide demonstrated strong associations. Emerging biomarkers such as high mobility group box 1, trimethylamine-N-oxide, and lipopolysaccharide showed high predictive potential but were supported by smaller cohorts. Conventional inflammatory markers, including C-reactive protein (CRP), high-sensitivity CRP, and interleukin, demonstrated weaker or inconsistent associations. Overall, biomarker performance varied by mechanistic category, study size, and timing of measurement.

**Conclusions:**

Multiple conventional and emerging biomarkers are associated with AF recurrence following catheter ablation with metabolic, remodeling, and natriuretic biomarkers demonstrating the strongest and more consistent predictive value. Integration of these biomarkers into clinical risk models may enhance prognostication. Further large-scale, standardized studies are required to validate their clinical utility and facilitate translation into routine practice.

## Introduction

Atrial fibrillation (AF) is not only the most common sustained cardiac arrhythmia but also represents a rapidly growing public health challenge. In the United States, estimates from large population-based projections suggest that the number of adults with diagnosed AF will increase substantially over the next decade, rising from approximately 2.7–6.1 million individuals currently affected to approximately 12.1 million by 2030 [1]. This projected rise reflects demographic aging, increasing prevalence of cardiovascular risk factors such as hypertension, diabetes, and obesity, and improved disease detection. AF is associated with markedly elevated risks of ischemic stroke, heart failure, hospitalization, and mortality, contributing substantially to cardiovascular morbidity and healthcare utilization [1–3].

The AF is driven by a complex interplay of comorbid conditions, underlying myocardial substrate, and structural and electrical remodeling, along with oxidative and pro-inflammatory stress affecting the myocardium [4–8]. Standard treatment of AF begins with antiarrhythmic medications to achieve either a rate or rhythm control, along with anticoagulation therapy to prevent embolic events [9]. Catheter ablation has become an established rhythm-control strategy, particularly in symptomatic patients and those with impaired ventricular function [10]. Early-stage catheter ablation has the potential to favorably modify the trajectory of AF by interrupting pathological remodeling processes responsible for progression from paroxysmal to persistent AF [11], thereby improving quality of life and decreasing morbidity and mortality [12–15].

Despite advances in ablation techniques, AF recurrence remains common, with reported rates ranging from 24% to 50% within the first year after the procedure [16–20]. Structural remodeling, atrial fibrosis, inflammation, and metabolic dysfunction contribute to AF persistence and recurrence [21–24]. However, traditional clinical risk scores (e.g., CHA_2_DS_2_-VASc, PAT_2_C_2_H, HATCH, APPLE, and CAAP-AF) demonstrate limited discriminatory performance for predicting post-ablation recurrence [25–28]. As a result, accurate identification of patients at high risk for recurrence before or after ablation remains an unmet clinical need.

Circulating biomarkers reflecting inflammation, myocardial remodeling, metabolic stress, and neurohormonal activation may offer incremental prognostic value beyond clinical parameters alone [29–36].

Although previous systematic reviews have examined discrete biomarker classes, such as inflammatory or natriuretic, most have focused on single biomarker classes or limited subsets of markers, rather than giving an integrated assessment of the vast variety of biomarkers linked with AF recurrence after catheter ablation. Additionally, several new biomarkers have recently emerged since earlier reviews like lipopolysaccharide (LPS), trimethylamine-N-oxide (TMAO), microRNAs, corin, etc. Therefore, this systematic review differs by providing a comprehensive and updated synthesis of both conventional and emerging biomarkers across multiple mechanistic pathways, incorporating recent metabolic indices (e.g., triglyceride glucose (TyG)-related markers, metabolic score for insulin resistance (METS-IR)), atrial-specific proteins (e.g., bone morphogenetic protein-10 (BMP-10)), gut microbiota-derived metabolites (e.g., TMAO), and molecular markers (e.g., microRNAs, corin, p63). Given its rising prevalence and associated adverse outcomes, improved strategies for risk stratification and recurrence prediction, such as the biomarker-based approaches reviewed herein, are critically needed to optimize patient outcomes. Additionally, we qualitatively stratified biomarkers according to strength of association and study size to provide a pragmatic perspective for clinical applicability.

## Methods

This systematic review was performed in accordance with Preferred Reporting Items for Systematic Reviews and Meta-analysis (PRISMA), 2020 guidelines [37]. Relevant studies were identified from major databases and eligible studies were assessed using quality appraisal tools followed by data extraction as described below.

### Search strategy and databases

To identify relevant articles, a comprehensive literature search was conducted from January 2020 to March 2025, across multiple databases including PubMed (using Medical Subject Headings (MeSH)), ScienceDirect, Cochrane Library, Europe PubMed Central, Google Scholar, and supplemented by scanning through bibliographies of articles. The search was restricted to above time period with focus on humans to capture contemporary biomarker research reflecting current ablation techniques, rhythm monitoring standards, and emerging molecular markers. We acknowledge that these restrictions may introduce selection bias and potentially exclude relevant earlier. The search was developed using key words such as “biomarkers,” “natriuretic peptides,” “atrial fibrillation recurrence,” and “catheter ablation.” A more detailed account of the search strategies, and the studies identified can be found in Table 1.

**Table 1:** Articles Identified Using Each Database

Database	Keywords	Search strategy	Filters	Search result
Pubmed MeSH/Medline/PMC	Biomarkers, natriuretic peptides, atrial fibrillation recurrence, catheter ablation	Biomarkers OR natriuretic peptides OR "Biomarkers/analysis"[Majr] OR "Biomarkers/blood"[Majr] OR "Biomarkers/chemistry"[Majr] OR "Biomarkers/metabolism"[Majr] AND atrial fibrillation OR atrial fibrillation recurrence OR "Atrial Fibrillation/complications"[Majr] OR "Atrial Fibrillation/diagnosis"[Majr] OR "Atrial Fibrillation/diagnostic imaging"[Majr] OR "Atrial Fibrillation/drug therapy"[Majr] OR "Atrial Fibrillation/etiology"[Majr] OR "Atrial Fibrillation/pathology"[Majr] OR "Atrial Fibrillation/physiopathology"[Majr] OR "Atrial Fibrillation/prevention and control"[Majr] AND catheter ablation OR "Catheter Ablation/adverse effects"[Mesh] OR "Catheter Ablation/instrumentation"[Mesh] OR "Catheter Ablation/methods"[Mesh]	Filters applied: in the last 5 years, English, humans, exclude preprints	79
Cochrane Library	Biomarkers, natriuretic peptides, atrial fibrillation recurrence, catheter ablation	"biomarkers" AND "atrial fibrillation" AND "catheter ablation"	Last 5 years, English	15
Science Direct	Biomarkers, natriuretic peptides, atrial fibrillation recurrence, catheter ablation	"Biomarkers" AND "atrial fibrillation recurrence" AND "catheter ablation"	Last 5 years, review articles, research articles	23
Europe PubMed Central		"Biomarkers" OR "natriuretic peptides" AND "atrial fibrillation recurrence" AND "catheter ablation"	Last 5 years, full text in Europe PMC, review articles, excluding preprints, books and documents	131
Google Scholar		“(“biomarkers” OR “natriuretic peptides”)” AND “atrial fibrillation recurrence” AND “catheter ablation”	Last 5 years	475

MeSH: Medical Subject Headings; PMC: PubMed Central.

### Inclusion and exclusion criteria

This systematic review included full-text articles in English published within the last 5 years from January 2020 to March, 2025 and focused on human subjects to ensure the accuracy, and relevance of the results. The selected articles specifically included biomarkers in AF recurrence with at least 1 year of follow-up after catheter ablation. Eligible study designs included prospective and retrospective observational cohort studies, case-control studies, and systematic reviews/meta-analyses evaluating biomarkers associated with AF recurrence after catheter ablation. Randomized controlled trials were eligible if biomarker analysis was reported.

Studies were excluded if they were published more than 5 years ago, lacked at least 1 year follow-up after catheter ablation, written in languages other than English, or based on non-human research to minimize bias and maintain the review’s focus. Additionally, conference abstracts, case reports, editorials, posters, preprints, presentations, and non-peer-reviewed studies were excluded.

### Selection process

All identified records were imported into EndNote (Clarivate, Philadelphia, PA), and duplicates were removed. Two independent reviewers screened titles and abstracts for eligibility based on predefined inclusion and exclusion criteria. Full-text articles of potentially eligible studies were then assessed independently by the same two reviewers. Disagreements at any stage were resolved through discussion and consensus; if consensus could not be reached, a third senior reviewer adjudicated.

### Quality appraisal and data extraction

Quality appraisal was conducted independently by two reviewers with prior training in systematic review methodology. Observational studies were assessed using Newcastle–Ottawa scale (NOS), and systematic reviews and meta-analyses were evaluated using Assessment of Multiple Systematic Reviews 2 (AMSTAR 2) as shown in Table 2 [38–64]. Inter-reviewer agreement for title/abstract screening and full-text eligibility assessment was quantified using Cohen’s kappa (κ). Agreement was substantial (κ = 0.82) for study selection and moderate-to-substantial (κ = 0.76) for quality appraisal. Discrepancies were resolved through discussion and consensus, with arbitration by a third reviewer when necessary. NOS scores were categorized as high quality (≥ 7), moderate quality (5–6), and low quality (< 5). AMSTAR 2 ratings followed established guidance (high, moderate, low, critically low confidence). Borderline scores (e.g., NOS 6) were interpreted conservatively as moderate quality and study was excluded after quality informed interpretative weighting. Quality scores did not determine inclusion but influenced interpretation strength. Risk-of-bias assessment informed the qualitative synthesis. Studies with higher methodological quality (NOS ≥ 7, or AMSTAR 2 moderate-to-high confidence) were given greater interpretative weight when summarizing strength of evidence.

**Table 2:** Quality Appraisal Tools Based on Their Study Design

Quality assessment tool	Type of study	Total score	Accepted score (> 70%)	Number of accepted studies
NCOT^1^	23 observational studies	9	7	Meyre et al 2020^1^ [38], Jaroonpipatkul et al 2023^2^ [39], Li et al 2022^1^ [40], AlKassas et al 2023^1^ [41], Wang et al 2021^1^ [42], Chen et al 2020^1^ [43], Li et al 2022^1^ [44], Hennings et al 2023^1^ [45], Tan et al 2021^1^ [46], Wei et al 2020^1^ [47], Li et al 2024^2^ [48], Shi et al 2024^2^ [49], El-Harasis et al 2024^1^ [50], Jia et al 2024^1^ [51], Tang et al 2022^1^ [52], Wang et al 2024^1^ [53], Gumanova et al 2024^1^ [54], Sustr et al 2024^1^ [55], Lage et al 2023^1^ [56], Zhao et al 2024^1^ [57], Meng et al 2024^1^ [58], Wang et al 2022^1^ [59], Kawaji et al 2023^1^ [60], Zhao et al 2023^1^ [61], Badoz et al 2021^1^ [62], Younes et al 2023^1^ [63], Yuan et al 2023^2^ [64]
AMSTAR2^2^	4 systematic review/meta-analysis	16	12	

NCOT: Newcastle–Ottawa Tool; AMSTAR 2: Assessment of Multiple Systematic Reviews 2.

Data extraction was performed independently by one reviewer and verified by a second reviewer to ensure accuracy and completeness. When studies evaluated multiple biomarkers, each biomarker was extracted and analyzed independently. However, study characteristics (sample size, follow-up duration, recurrence rates) were counted once per study to avoid duplication. Biomarkers were synthesized qualitatively, and no pooled effect sizes were calculated across biomarkers.

Data extraction included: study title, first author, publication year, study design, sample size, mean age, sex distribution, AF subtype (paroxysmal/persistent), comorbidities, type of ablation procedure, biomarker(s) evaluated, timing of biomarker measurement (pre- or post-ablation), follow-up duration, definition of AF recurrence, rhythm monitoring modality, recurrence rate, effect estimates (hazard ratio (HR), odds ratio (OR), standardized mean difference (SMD)), confidence intervals (CIs), and covariate adjustments.

Biomarkers were categorized as “conventional” if they are widely established in cardiovascular clinical practice or have been extensively validated in AF or heart failure (e.g., C-reactive protein (CRP), high-sensitivity C-reactive protein (hs-CRP), B-type natriuretic peptide (BNP), N-terminal pro-B-type natriuretic peptide (NT-proBNP), interleukin-6 (IL-6)). Biomarkers were classified as “emerging” if they represent novel molecular markers, mechanistic candidates, metabolic indices, or biomarkers with limited large-scale validation and primarily recent evidence (e.g., BMP-10, suppression of tumorigenicity 2 (sST2), TyG-related indices, TMAO, corin, microRNAs).

Lastly, in order to facilitate interpretation of the evidence, we did a structured qualitative assessment of biomarkers based on commonly used prognostic principles in descriptive framework, such as adjusted effect estimates, effect magnitude, discrimination (area under the curve (AUC) where available), consistency across studies, and precision of intervals, and this assessment was not intended as a validated scoring system.

### Outcomes and follow-up

The primary outcome was late AF recurrence, defined as any documented atrial arrhythmia (AF, atrial flutter, or atrial tachycardia lasting ≥ 30 s) occurring after a 3-month post-ablation blanking period, in accordance with contemporary consensus definitions. Early arrhythmia occurring within the first 3 months post-ablation was classified as part of the blanking period and was not considered recurrence. Rhythm monitoring was performed using 12-lead electrocardiogram (ECG), 24-h Holter monitoring, extended ambulatory monitoring, implantable loop recorders, or validated handheld devices at scheduled follow-up intervals (typically 1, 3, 6, and 12 months or longer).

## Results

**Figure 1:**
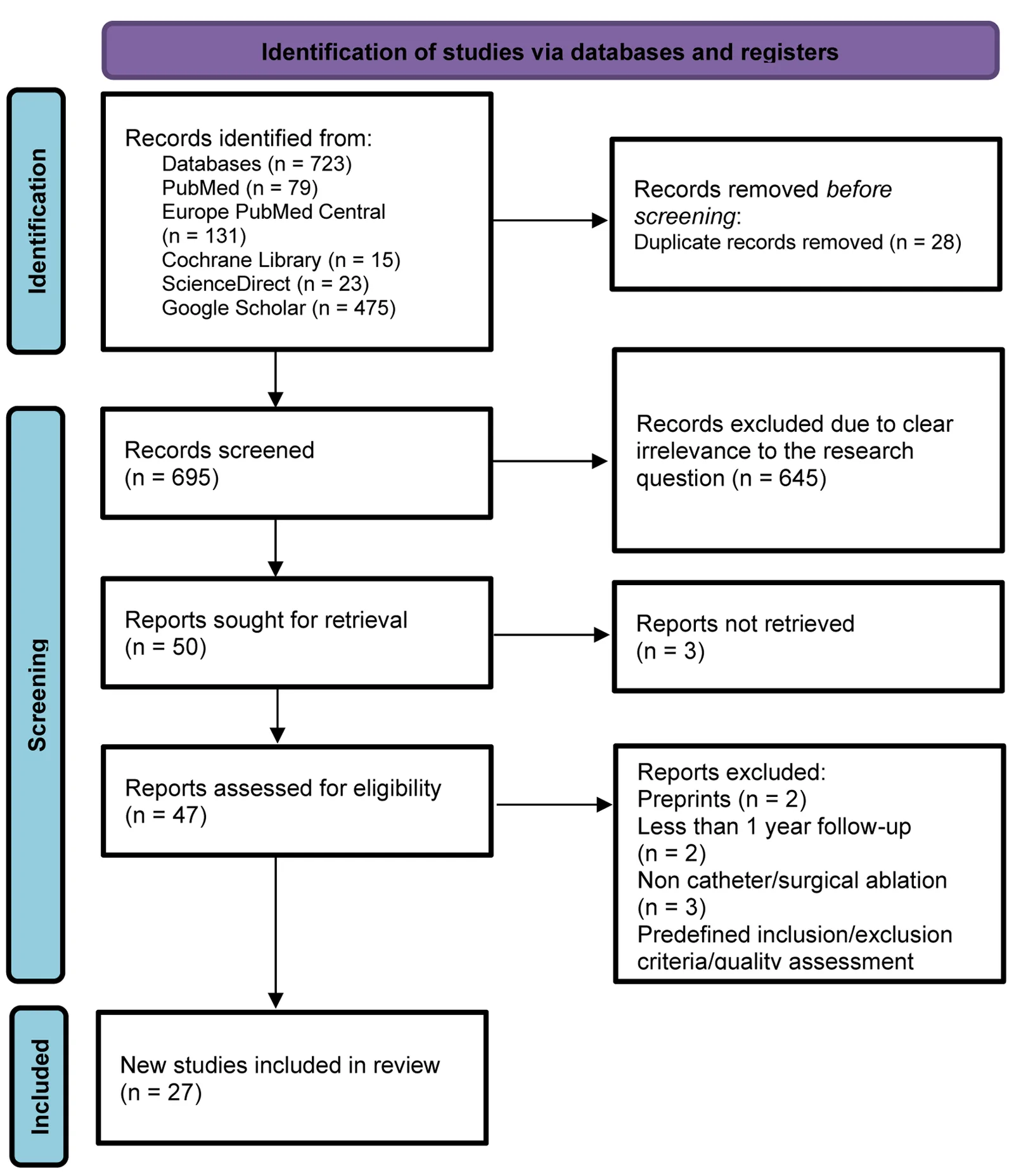
PRISMA flow diagram.

A total of 723 records were identified through database searches (PubMed, Europe PubMed Central, ScienceDirect, Cochrane Library, and Google Scholar). After removal of 28 duplicates, 695 records were screened by title and abstract, resulting in the exclusion of 645 articles. Fifty full-text articles were assessed for eligibility, of which three were excluded based on predefined inclusion and exclusion criteria. Consequently, 47 studies underwent quality appraisal, and 27 articles were included in the final systematic review. The study selection process is presented in the PRISMA flow diagram (Fig. 1).

### Study characteristics

Our systematic review included 27 observational studies, systematic reviews and meta-analysis published between January 2020 and March 2025, with most studies conducted in China. Collectively, these studies included 26,702 participants, with individual study sizes ranging from 42 to 10,085 participants. The mean age of participants across the included studies ranged from 53.24 to 71.3 years, with a predominance of males (approximately 44.2–78.1% males versus 23–48.7% females). The most frequently reported comorbidities were hypertension, dyslipidemia, prior stroke or transient ischemic attack (TIA), alcohol use, diabetes mellitus, smoking, and coronary artery disease. Average follow-up duration varied from 12 to 36 months. Across studies, most patients underwent first time AF ablation. Common exclusion criteria across studies were acute coronary syndrome, structural heart disease, congenital heart disease, valvular heart disease, severe heart failure, severe liver disease, severe kidney diseases, chronic inflammatory diseases, and acute infections.

Across included studies, paroxysmal AF predominated in approximately 55–65% of cohorts, with persistent/permanent AF comprising the remainder. Subtype-specific analyses were reported when available; however, subgroup findings were not pre-specified and should be interpreted cautiously. The ablation approach was predominantly based on pulmonary vein isolation, often complemented by adjunctive procedures, including superior vena cava isolation and additional ablations. The overall AF recurrence rates varied from 15.75% to 51%. The biomarker reliability grading criteria were done as shown in Table 3, and the key findings for the evaluated biomarkers, stratified by mechanistic category, are summarized in Tables 4 and 5.

**Table 3:** Grading Criteria Used for Biomarker Reliability

Strong reliability	Moderate reliability	Weak reliability
Adjusted HR/OR ≥ 1.8 Statistically significant (P < 0.05) Multivariable adjustment performed AUC ≥ 0.75	Adjusted HR/OR 1.3–1.79 Statistically significant AUC 0.65–0.74 Independent predictor but moderate effect size	Only mean difference Borderline effect No multivariable adjustment AUC < 0.65 Inconsistent results

AUC: area under the curve; HR: hazard ratio; OR: odds ratio.

**Table 4:** Core Mechanistic Biomarkers of Post-Ablation AF Recurrence (Inflammatory, Remodeling, Metabolic)

Biomarker	Timing	Adjusted effect	AUC	Design	N	AF type	Follow-up methods	Strength	Reliability
Inflammatory biomarkers									
CRP [38]	Pre	aHR 1.60 (Q4); 1.21 per unit	-	Prospective	711	Paroxy AF	ECG, Holter	Moderate dose-response	Moderate
hs-CRP [39]	Post	WMD 0.09	-	Meta-analysis	789	AF	ECG, Holter	Small mean difference	Weak
HMGB1 [40]	Post	OR 5.29; HR 3.81 for AF free survival	0.68	Prospective	72	Paroxy AF	ECG, Holter	Large effect size, limited sample	Strong
TNF-α, visfatin, adiponectin [41]	Pre	β 0.28, β 0.35, β 0.30	0.67, 0.73, 0.70	Prospective	117	Paroxy/persist AF	ECG, Holter	Independent predictor	Moderate-strong
IL-6, angiopoietin-2 [50]	Pre	OR 1.02, OR 1.08	0.71, 0.71	Retrospective	1,873	Paroxy/persist AF	ECG, Holter	Independent (small effect)	Weak-moderate
CA-125 [42]	Pre	aHR 4.25 (Q4); 3.23 per unit	-	Prospective	353	AF	ECG, Holter	Large graded effect; strong dose-response	Strong
MHR [43]	Pre	OR 1.34	0.71	Retrospective	125	Paroxy AF	ECG, Holter	Moderate independent effect	Moderate
Remodeling biomarkers									
TIMP-1 [44]	Pre	aHR 1.80 high vs. low	-	Prospective	194	Paroxy/persist AF	ECG, Holter	Independent/moderate effect	Moderate
BMP-10 [45]	Pre	aHR 1.98 per unit	-	Prospective	1,112	Paroxy/persist AF	ECG, Holter	Near 2-fold risk	Strong
sST2 [46]	Pre	HR 1.04 high vs. low (persistent AF)	0.75	Prospective	210	Paroxy/persist AF	ECG, Holter	Good discrimination	Moderate-strong
GDF-15 [47]	Pre	HR 1.053 (recurrent group)	-	Prospective	150	Persist AF	ECG, Holter	Modest effect	Moderate
Homocysteine [48]	Pre	OR 2.75, I^2^ = 0% (high vs. low)	-	Meta-analysis	2,147	AF	ECG, Holter	Highly consistent, I^2^ = 0%	Strong
sST2 [49]	Pre	SMD 1.15, I^2^ = 92% (recurrent group)	-	Meta-analysis	1,779	AF	ECG, Holter	Significant heterogeneity	Moderate
Metabolic biomarkers									
TyG index [51]	Pre	HR 2.06 (high vs. low), HR 1.58 (locus 3)	-	Retrospective	997	Paroxy/persist AF	24 ambulatory ECG	Consistent	Strong
TyG index [52]	Pre	aHR 2.02	0.74	Retrospective	275	Paroxy/persist AF	ECG, Holter	Independent	Strong
METS-IR [53]	Pre	aHR 1.82 (tertile 3), 1.04 per unit	-	Retrospective	2,242	Paroxy/persist AF	ECG, Holter	Graded risk, large sample	Moderate-strong
TyG-BMI	Pre	aHR 1.71 (tertile 3), 1.01 per unit	-					Independent	Moderate-strong
TyG index	Pre	aHR 1.25 (tertile 3), 1.18 per unit	-					Independent	Moderate
TG/HDL-C	Pre	Not significant (P > 0.05)	-					No predictive value	Not reliable

CRP: C-reactive protein; hs-CRP: high-sensitivity C-reactive protein; HMGB1: high mobility group box 1; TNF-α: tumor necrosis factor-α; CA-125: plasma carbohydrate antigen-125; MHR: monocyte/high-density lipoprotein ratio; TIMP-1: tissue inhibitor of metalloproteinase-1; BMP10: bone morphogenetic protein 10; sST2: soluble suppression of tumorigenicity 2; GDF-15: growth differentiation factor 15; Hcy: homocysteine; IL-6: interleukin 6; TyG: triglyceride glucose; METS-IR: metabolic score for insulin resistance; TyG-BMI: triglyceride glucose body mass index; TG/HDL-C: triglyceride to high-density lipoprotein cholesterol; HR: hazard ratio; OR: odds ratio; AUC: area under the curve; pre: pre-ablation; post: post-ablation; WMD: weighted mean difference; SMD: standardized mean difference.

**Table 5:** Natriuretic Peptides and Other Emerging Biomarkers of Post-Ablation AF Recurrence

Biomarker	Timing	Adjusted effect	AUC	Design	N	AF type	Follow-up methods	Strength	Reliability
Other biomarkers									
RAD51/p63 [54]	Pre	Mean difference only	-	Prospective	146	Paroxy/persist AF	ECG, Loop recorder	Mean difference only	Weak
miR-206 [55]	Pre	OR 2.65 per unit	-	Prospective	49	Paroxy/persist AF	ECG, Holter	Small sample	Moderate
miR-451a [56]	Post	-	0.72	Prospective	42	Paroxy/persit AF	ECG, Holter	Small sample, no aHR	Moderate
Corin [57]	Pre	aHR 1.46, > 494.85 pg/mL	0.68	Prospective	616	Paroxy/persist AF	ECG, Holter	Moderate discrimination	Moderate
TMAO [58]	Pre	HR 13.53 for cutoff > 4.3 µM serum level	0.84	Prospective	152	Paroxy/Persist AF	ECG, Holter	Highest discrimination	Very strong
LPS [59]	Pre	HR 5.69 Log LPS	-	Retrospective	159	AF	ECG, Holter	Large effect (wide CI)	Strong
Natriuretic peptides									
BNP [60]	Pre	aHR 0.63 for lower AF risk with low BNP	-	Retrospective	1,750	Paroxy/persit AF	ECG, Holter		Moderate
NT-proBNP [61]	Pre	HR 2.89 for NT-proBNP > 168.05 pg/mL	0.68	Retrospective	326	Paroxy/persist AF	ECG, Holter	Independent	Strong
MR-proANP [62]	Pre	OR 24.27 for MRproANP > 107.9 pmol/L	-	Prospective	105	Paroxy/persit AF	ECG, Holter	Extremely large independent effect	Very strong
BNP [63]	Pre	aHR 1.85 per 100 unit	-	Retrospective	125	Persist AF	1 lead EKG smartphone device	Reproducible	Moderate-strong
ANP/BNP/MR-proANP/NT-proBNP) [64]	Pre	SMD 0.39, I^2^ 45% for ANP, 0.51 I^2^ 93% for BNP, 0.71 I^2^ 84% for NT-proBNP, 0.91 I^2^ 88% for MR-proANP	-	Meta-analysis	10,085	Paroxy/persist AF	ECG, Holter	High heterogeneity	Moderate-variable

miR: microRNAs; TMAO: trimethylamine-N-oxide; LPS: lipopolysaccharide; BNP: B-type natriuretic peptide; NT-proBNP: N-terminal pro-B-type natriuretic peptide; MR-proANP: mid-regional N-terminal pro-atrial natriuretic; ANP: atrial natriuretic peptide; HR: hazard ratio; OR: odds ratio; AUC: area under the curve; pre: pre-ablation; post: post-ablation; SMD: standardized mean difference.

Observational cohort studies contributed primary data for biomarker-outcome associations. Systematic reviews and meta-analyses were used to contextualize findings but were not pooled alongside primary studies to avoid duplication of evidence. When overlap between primary studies and meta-analysis was identified, only the primary study data were considered in biomarker-level synthesis. Biomarker timing also varied substantially (baseline pre-ablation vs. immediate post-procedural vs. delayed follow-up sampling), which may influence measured levels, particularly for inflammatory markers sensitive to procedural injury.

A formal quantitative meta-analysis was not performed due to substantial clinical and methodological heterogeneity across studies, including differences in biomarker assays, effect size reporting (HR, OR, SMD), AF subtype composition, follow-up duration, and statistical adjustment models. Therefore, a qualitative synthesis was undertaken and formal assessment of publication bias (e.g., funnel plot analysis, or Egger testing) was not performed because a quantitative meta-analysis was not conducted. Additionally, selective outcome reporting at the individual study level was not systematically evaluated beyond quality appraisal tools. Selective reporting remains a potential limitation in biomarker-heavy literature.

## Discussion

**Figure 2:**
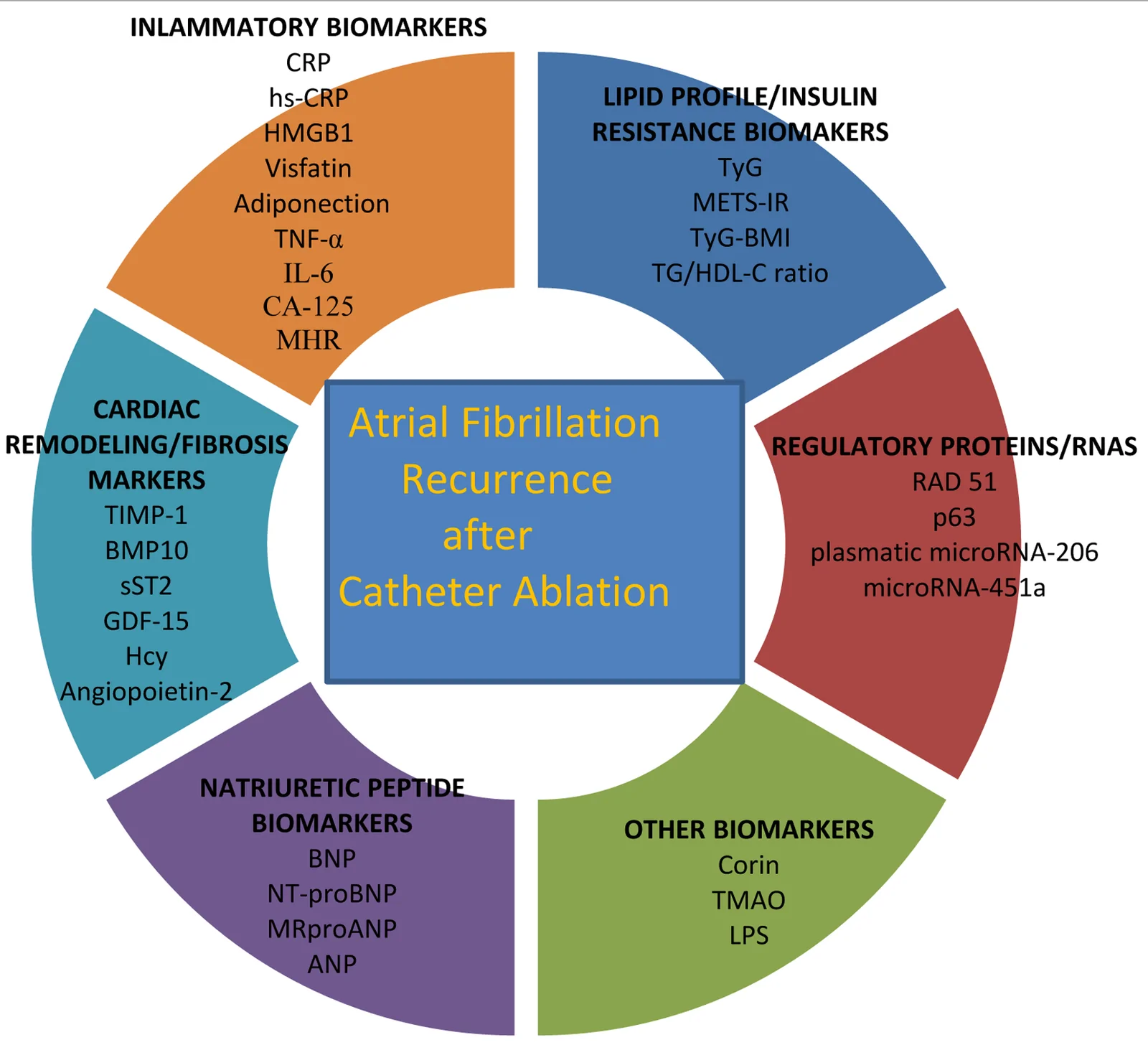
Biomarker subgroups associated with post-ablation AF recurrence. CRP: C-reactive protein; hs-CRP: high-sensitivity C-reactive protein; HMGB1: high-mobility group box 1; TNF-α: tumor necrosis factor-α; CA-125: carbohydrate antigen-125.

This systematic review included 27 studies that examined biomarkers in relation to AF recurrence after catheter ablation and identified 30 conventional and novel biomarkers. These biomarkers have been associated with various mechanisms involved in AF pathogenesis, including inflammation, oxidative stress, cardiac remodeling, fibrosis, lipid profile-insulin resistance indexes, natriuretic peptides, and other molecular pathways [29–35], as illustrated in Figure 2.

The classification of biomarkers as conventional versus emerging was based on prior clinical validation, extent of integration into routine cardiovascular practice, and the maturity of available evidence as shown in Table 6.

**Table 6:** Conventional and Emerging Biomarkers Associated With AF Recurrence Post-Ablation

Emerging biomarkers for AF recurrence after catheter ablation	Conventional biomarkers for AF recurrence after catheter ablation
HMGB1, visfatin (adipocytokine), adiponectin, plasma CA-125, MHR, TIMP-1, BMP-10, sST2, GDF-15, RAD51 & p63, plasmatic microRNA-206, miRNAs/miR-451a, corin, serum Hcy, angiopoietin-2, TyG index, METS-IR, TyG-BMI, TMAO, serum LPS, TG/HDL-C ratio	CRP), hs-CRP, TNF-α, IL-6, BNP, MR-proANP, ANP), NT-proBNP

To facilitate interpretation of the evidence, biomarkers were qualitatively categorized according to predefined prognostic characteristics rather than a validated scoring system. The assessment considered, as mentioned in Table 3, below ranking criteria and is presented in Table 7. Conclusions regarding biomarker strength were informed by methodological quality, consistency across cohorts, and adjustment for confounders. Observational design and residual confounding limit causal inference.

**Table 7:** Structured Qualitative Assessment of Biomarker Predictive Strength and Reliability

Very strong associations	Strong associations	Moderate to moderate-strong associations	Weak associations/limited evidence
MR-proANP (OR 24.27) TMAO (HR 13.53, AUC 0.84) CA-125 (aHR 4.25 Q4)	HMGB1 (OR 5.29) NT-proBNP (HR 2.89) TyG Index (HR 2.06) BMP-10 (aHR 1.98) LPS (HR 5.69) BNP-elevated (aHR 1.85)	sST2 in persistent AF (HR 1.04/AUC 0.75), visfatin (β 0.35/AUC 0.73), METS-IR (aHR 1.82) TyG-BMI (aHR 1.71) TIMP-1 (aHR 1.80) Corin (aHR 1.46/AUC 0.68) MHR (OR 1.34/AUC 0.71) TNF-α (β 0.28/AUC 0.67) Adiponectin (β 0.30/AUC 0.70) Angiopoietin-2 (OR 1.08/AUC 0.71) CRP (aHR 1.60)	IL-6 (minimal OR 1.02) RAD51/p63 (mean difference only) miR-206 (small sample 49) miR-451a (small sample/no adjusted HR) GDF-15 (HR 1.05, small effect)

CRP: C-reactive protein; hs-CRP: high-sensitivity C-reactive protein; HMGB1: high mobility group box 1; TNF-α: tumor necrosis factor-α; CA-125: plasma carbohydrate antigen-125; MHR: monocyte/high-density lipoprotein ratio; TIMP-1: tissue inhibitor of metalloproteinase-1; BMP-10: bone morphogenetic protein 10; sST2: soluble suppression of tumorigenicity 2; GDF-15: growth differentiation factor 15; Hcy: homocysteine; IL-6: interleukin 6; TyG: triglyceride glucose; METS-IR: metabolic score for insulin resistance; TyG-BMI: triglyceride glucose body mass index; TG/HDL-C: triglyceride to high-density lipoprotein cholesterol; miR: microRNAs; TMAO: trimethylamine-N-oxide; LPS: lipopolysaccharide; BNP: B-type natriuretic peptide; NT-proBNP: N-terminal pro-B-type natriuretic peptide; MR-proANP: mid-regional N-terminal pro-atrial natriuretic; ANP: atrial natriuretic peptide; HR: hazard ratio; OR: odds ratio; AUC: area under the curve.

The ranking criteria included: priority to adjusted HR/OR, effect magnitude, AUC (≥ 0.75 strong discrimination), dose-response relationship, and precision of CI.

### Overview of the main findings

A substantial proportion of included cohorts originated from East Asia, particularly China. This geographic concentration may limit generalizability due to differences in AF phenotype, comorbidity burden, genetic background, and healthcare systems. Timing of biomarker assessment varied substantially. Pre-ablation measurements likely reflect underlying atrial substrate and systemic burden, whereas immediate post-procedural elevations (particularly inflammatory markers) may reflect ablation-related myocardial injury. Differences in sampling timing may partially explain inconsistent findings for hs-CRP, Angiopoietin-2 (Ang2), and sST2 across studies.

Several biomarkers may intersect with established biological pathways. For example, corin is directly involved in activation of natriuretic peptides through cleavage of pro-atrial natriuretic peptide, linking it mechanistically to atrial stretch signaling. Microbiota-derived metabolites such as TMAO, and LPS may promote systemic inflammation and oxidative stress, which contribute to atrial remodeling and arrhythmogenic substrate formation.

Considerable heterogeneity existed in assay platforms, laboratory calibration standards, and reported thresholds. For example, hs-CRP and carbohydrate antigen-125 (CA-125) cutoffs varied widely across cohorts, and natriuretic peptide measurements were performed using different immunoassays. Most studies evaluating emerging biomarkers were single-center investigations with relatively small sample sizes. This variability limits direct comparability across studies and may hinder clinical translation.

Heart failure status was variably reported across studies. In cohorts with a higher prevalence of structural heart disease, or reduced ventricular function, natriuretic peptides (BNP, NT-proBNP, mid-regional pro-atrial natriuretic peptide (MR-proANP)) demonstrated stronger associations with recurrence risk. However, what these associations reflect, atrial-specific stretch versus concomitant ventricular dysfunction, remains uncertain.

AF phenotype and comorbidity burden likely influence biomarker performance. Persistent AF, characterized by greater atrial fibrosis and remodeling, may amplify associations with fibrosis-related biomarkers such as BMP-10 and sST2. Conversely, natriuretic peptide levels may be influenced by underlying ventricular dysfunction, particularly in heart failure population, underscoring the need for careful adjustment for ventricular function and structural parameters when interpreting their prognostic significance.

Most available evidence linking circulating biomarkers with AF recurrence after catheter ablation derives from observational associations. Although several biomarkers demonstrated significant adjusted associations with recurrence risk, formal evaluation of incremental predictive value beyond established clinical and echocardiographic predictors remains limited. Among the biomarkers reviewed, natriuretic peptides (NT-proBNP, MR-proANP), metabolic markers such as the TyG index and homocysteine (Hcy), and selected remodeling markers (e.g., BMP-10, CA-125) appear most promising for near-term clinical validation due to stronger and more consistent associations across studies. In contrast, emerging biomarkers like LPS, TMAO, and high mobility group box 1 (HMGB1) while showing a stronger association should currently be considered exploratory given limited sample sizes and lack of external validation. Inflammatory markers (e.g., tumor necrosis factor (TNF)-alpha, adiponectin, Ang2, hs-CRP) showed more variable and generally modest associations across AF subtypes. Future research should prioritize prospective biomarker-guided prediction models incorporating standardized assays, predefined thresholds, and rigorous evaluation of clinical utility through decision-analytic frameworks before routine clinical adoption can be recommended.

Finally, compared with prior reviews that focused on individual biomarker classes, this study provides a complete and contemporary synthesis of 30 conventional and new biomarkers covering mechanistic pathways including inflammatory, remodeling, metabolic, natriuretic, and molecular mechanisms. By combining recent evidence and methodically assessing the strength and consistency of reported associations, our findings offer a realistic methodology for choosing biomarkers for further validation and integration into multimarker risk prediction models.

### Inflammatory biomarkers for prediction of AF recurrence after catheter ablation

Both epidemiological and histological evidence suggests that inflammation significantly contributes to the onset and persistence of AF [65, 66]. The inflammatory biomarkers included in our systematic review are shown in Figure 2.

A prospective cohort study conducted by Meyre et al demonstrated that elevated pre-ablation CRP levels were associated with a higher risk of AF recurrence after catheter ablation [38]; however, this study excluded patients with long standing persistent or permanent AF. Supporting this observation, several earlier studies have also identified a potential association between elevated CRP levels and AF recurrence following catheter ablation [67, 68]. These earlier studies, however were limited by small sample sizes and retrospective design, whereas the larger cohort studied by Meyre et al [38] provided more robust validation.

Baseline hs-CRP levels did not differ significantly between patients with and without AF recurrence; however, post-ablation hs-CRP levels were significantly higher in the recurrence group, according to a meta-analysis by Jaroonpipatkul et al, which included 789 subjects across 10 studies [39]. In a more recent meta-analysis by Boyalla et al [36], higher baseline hs-CRP levels were observed in patients with AF recurrence compared with those without recurrence following ablation. However, the included studies showed high heterogeneity, and upon removal of outliners, the heterogeneity reduced to a moderate, accompanied by a decreased strength of association between hs-CRP and AF recurrence.

HMGB1 is a protein secreted by necrotic cells that has been shown to initiate inflammatory responses [69]. Following catheter ablation, HMGB1 expression has been found to increase, contributing to sustained oxidative stress and ongoing inflammation [70]. This was demonstrated in a study by Li et al [40], which reported higher post-ablation levels of HMGB1 and their association with AF recurrence, showing moderate predictive power. However, this study included only patients with paroxysmal AF and had a small sample size. Therefore, HMGB1 could serve a potential predictor reflecting inflammation and the extent of myocardial injury if validated in larger studies.

Visfatin is an adipocytokine secreted by adipose tissue and plays a role in promoting inflammation and fibrosis. Elevated pre-procedural levels of visfatin and TNF-α were found to predict AF recurrence after ablation with an accuracy of 50% and 34%, respectively, as shown in a study by AlKassas et al [41]. These findings are further supported by the study by Plantek et al [71], which also identified an association between higher visfatin levels and AF recurrence. If validated in larger cohort, visfatin could serve as a reliable predictor of AF recurrence after catheter ablation.

CA-125 is a circulating soluble glycoprotein expressed by the coelomic epithelium and released in response to mechanical stress and inflammatory processes [72]. Higher pre-ablation CA-125 levels were higher in AF recurrence group than in non-recurrence group and were shown to independently predict AF recurrence after radiofrequency catheter ablation (RFCA), as demonstrated in the study by Wang et al [42]. This finding was supported by the study by Huang et al [73]; however, they used cut-off value of 11.05 U/mL against 13.75 U/mL in the former study. Post-ablation CA-125 levels were not assessed and if these results are validated in larger cohorts, CA-125 could become a valuable marker of AF recurrence after catheter ablation.

The higher pre-ablation monocyte-to-high-density-lipoprotein-cholesterol (HDL) ratio (MHR) was found associated with an increased risk of AF and may predict recurrence after catheter ablation, as validated in a study by Canpolat et al [74]. This was consistent with our included study by Chen et al [43], which showed elevated pre-ablation MHR to be linked with higher risk of AF recurrence following ablation. However, the study was limited by a modest sample size and should be interpreted cautiously when generalizing these findings.

### Myocardial remodeling/fibrosis biomarkers for prediction of AF recurrence after catheter ablation

Cardiac remodeling, characterized by collagen deposition and fibrosis, has been implicated in the pathogenesis of AF as well as in the recurrence of arrhythmias after catheter ablation [75, 76]. The cardiac remodeling and fibrosis biomarkers included in our systematic review are presented in Figure 2.

BMP-10, belonging to the transforming growth factor-β superfamily, serves as a biomarker specific to atrial tissue and is released into the bloodstream during atrial development and structural remodeling [77–79]. The study by Hennings et al [45] included in our review demonstrated that BMP-10, a novel atrial specific biomarker, demonstrated a strong association with AF recurrence after catheter ablation, with a large patient cohort supporting the strength of this association. This observation is further supported by a study by Reyat et al [80], which identified BMP-10 as the strongest predictor for AF recurrence following catheter ablation. It is suggested that elevated BMP-10 levels may reflect a more advanced stage of atrial cardiomyopathy, a condition associated with increased risks of AF and stroke. However, the precise mechanisms underlying BMP-10’s role in this process are still not fully understood [81].

Hcy, through mechanisms involving endothelial dysfunction, oxidative stress, inflammation, and thrombogenesis, has been linked to various cardiovascular conditions [82, 83] as well as atrial remodeling and changes in electrophysiological properties [84–86]. Emerging evidence suggests that elevated serum Hcy levels may contribute to the development of AF [87]. In a meta-analysis by Li et al [48], higher pre-ablation Hcy levels were linked to an increased risk of AF recurrence after ablation. Although this association was well established (P < 0.001, I^2^ = 0%), the meta-analysis predominantly included observational studies from China, which may limit the generalizability of the findings. If findings are validated in larger randomized clinical trials, these findings could have significant impact on reducing AF recurrence by lowering Hcy levels through folic acid administration, as demonstrated in a study by Dong et al [88].

Ang2 is an endothelial growth factor with pro-inflammatory properties that influences angiogenesis depending on vascular endothelial growth factor (VEGF) [89]. In included study by El-Harasis et al [50], Ang2 and IL-6 were associated with AF recurrence after catheter ablation among nine other biomarkers studied. This study had a large cohort of patients compared to previous others, such as Reyat et al [80], which showed no association with Ang2 and AF recurrence after catheter ablation in a sample of 359 patients. Another study with 187 patients reported higher baseline Ang2 levels in AF recurrence group on univariate analysis, although minimal increase in AF recurrence was seen on multivariate analysis [90]. The significant association of IL-6 with AF recurrence observed in the included study aligns with prior studies [91–95] and a recent meta-analysis [36], although the sample sizes in these studies were relatively small.

sST2 is a soluble isoform of the Toll-like/IL-1 receptor family that binds interleukin-33 (IL-33), preventing its interaction with the transmembrane isoform ST2L [96–98]. This inhibition prevents IL-33 from exerting its protective effects, thereby promoting inflammation and fibrosis [99]. In the included study by Tan et al [46], sST2 was identified as a marker associated with AF recurrence in patients with persistent AF following ablation, but not in those with paroxysmal AF. In contrast, a recent met-analysis by Shi et al [49] in our review study showed higher pre-ablation levels of sST2 in the recurrent AF group. Notably, studies with ≥ 60% paroxysmal AF showed a greater difference in serum sST2 levels between recurrence and non-recurrence groups than studies with < 60%. This variation may be due to small sample size of the former study and possibly a higher and more frequent AF burden in paroxysmal AF group. Future larger studies are needed to validate these findings.

The accumulation of extracellular matrix leading to atrial fibrosis is governed by the interplay between matrix metalloproteinases (MMPs) and tissue inhibitors of metalloproteinases (TIMPs) [21, 75]. Nair and Gongora found a positive correlation between plasma growth differentiation factor 15 (GDF-15) concentrations and the levels of MMPs and TIMIPs [100]. Tissue inhibitor of metalloproteinase-1 (TIMP-1) and GDF-15 serves as a marker of myocardial fibrosis and cardiac remodeling, which increase with a higher arrhythmia burden [101–103]. In the included study by Li et al [44], pre-ablation TIMP-1 levels were higher in the AF recurrence group after catheter ablation and were predictive of AF recurrence after RFCA. Similarly, another included study by Wei et al [47] reported that increased pre-procedural GDF-15 levels were associated with AF recurrence after catheter ablation. Although both studies had modest sample sizes, validation in larger cohorts could establish these markers as useful tools for pre-ablation risk stratification.

### Metabolic/insulin resistance biomarkers for prediction of AF recurrence after catheter ablation

The insulin resistance biomarkers included in our systematic review are shown in Figure 2. The TyG index, METS-IR, triglyceride glucose–body mass index (TyG-BMI), and triglyceride to high-density lipoprotein cholesterol (TG/HDL-C) ratio represent accessible and robust indices for the assessment of insulin resistance [104]. Insulin resistance (IR) is known to cause cardiac lipotoxicity, oxidative stress damage, abnormal intracellular calcium homeostasis, hyperphosphorylation of calcium-related proteins, and cardiac fibrosis, all of which contribute to structural remodeling of atria and increased AF risk [105–107]. IR independently predicts AF recurrence after catheter ablation in non-diabetic patients [108], while AF recurrence rates are elevated in those with diabetes [109]. In a large retrospective observational study by Wang et al [53] in our review, the TyG index, METS-IR, and TyG-BMI were independently associated with post-ablation AF recurrence, with TyG-BMI demonstrating strongest predictive value, followed by METS-IR. Similarly, Jia et al [51] reported a significant relationship between the TyG index and AF recurrence following ablation. In addition, Tang et al [52] observed that higher pre-ablation TyG index levels were associated with an increased risk of subsequent AF recurrence after RFCA in non-diabetic patients.

### Natriuretic peptide biomarkers for prediction of AF recurrence after catheter ablation

Natriuretic peptides are well-established biomarkers in patients with heart failure, and their significance has also been recognized in AF [110–112]. In several cohorts, BNP and NT-proBNP retained independent associations with AF recurrence after adjustment for age, AF type, left atrial diameter, and selected clinical covariates. However, only a minority of studies formally evaluated incremental predictive value beyond structural echocardiographic parameters using C-statistic or reclassification analyses. Where assessed, improvements were modest, suggesting that natriuretic peptides may provide complementary rather than transformative prognostic enhancement.

In our systematic review (shown in Fig. 2), the study by Kawaji et al [60] found that low BNP concentrations were associated with lower rates of recurrent atrial tachyarrhythmias after catheter ablation in patients with paroxysmal AF only, compared to patients with higher BNP levels and heart failure. This study included a sizable cohort of 1,750 patients. One possible explanation of low BNP levels is a lesser arrhythmic burden in paroxysmal AF patients. In addition to cardiac hemodynamic changes, higher levels of BNP and NT-proBNP have been associated with the progression of AF and the presence of non-paroxysmal AF [113–116].

A meta-analysis by Yuan et al [64] demonstrated that elevated baseline levels of natriuretic peptides, including ANP, BNP, NT-proBNP, and mid-regional N-terminal pro-atrial natriuretic peptide (MR-proANP), were associated with an increased risk of AF recurrence following catheter ablation. Similarly, MR-proANP independently predicted recurrent AF in a study by Badoz et al [62], and high pre-ablation NT-proBNP levels were associated with AF recurrence following RFCA in a study by Zhao et al [61].

In literature, atrial volume changes show a modest correlation with BNP levels [117–119], and possible ventricular dysfunction due to tachyarrhythmias may also contribute to increased BNP levels. A study by Younes et al [63], which included 125 patients without heart failure, found that the recurrent AF group had higher pre-ablation BNP levels, and BNP was a significant predictor of AF recurrence following catheter ablation in patients with persistent AF. Besides mechanistic reasons mentioned above, inflammatory processes have been shown to elevate BNP levels and up-regulate its gene expression in cardiac tissue [120].

### Other biomarkers for prediction of AF recurrence after catheter ablation

Circulating soluble corin expressed as a membrane bound serine protease primarily on cardiomyocytes and responsible for activating cardiac natriuretic peptides, was found to be elevated and served as a valuable predictor for AF recurrence after catheter ablation in a study by Zhao et al [57], which included 616 patients. If validated in future studies, it could serve as a potential biomarker for predicting AF recurrence following catheter ablation.

The gut microbiota-derived metabolites, TMAO and LPS, were found elevated in recurrent AF group after catheter ablation in studies by Meng et al [58] and Wang et al [59], respectively. Patients with persistent AF exhibited significantly higher serum TMAO concentrations than individuals in sinus rhythm or with paroxysmal AF. Circulating LPS may contribute to recurrent AF by increasing systemic inflammation and atrial fibrosis.

Increased levels of p63 protein and RAD51, which are involved in genome stability and DNA repair, have been associated with AF recurrence after catheter ablation in a study by Gumanova et al [54]. These proteins may contribute to structural impairment of the atria. However, the relatively small cohort and limited number of AF recurrence events after 1 year of follow-up may have affected the robustness of the findings.

MicroRNAs constitute a group of non-coding RNAs that control gene expression through interference with mRNA transcription and thereby affecting final protein levels, and have been implicated in the development and recurrence of AF. Sustr et al [55] reported significantly elevated levels of circulating microRNA-206 in patients with early AF recurrence. Similarly, Lage et al [56] found that microRNA-451a expression was down-regulated in individuals with recurrent AF. While microRNA expression appears to be associated with AF recurrence, the limited sample sizes of the available studies emphasize the need for larger scale research to validate their utility as biomarkers of AF recurrence.

### Limitations

Several limitations must be acknowledged. First, most of the included studies were predominantly observational, which limits the ability to infer causality between biomarkers and AF recurrence. Considerable heterogeneity existed across studies in terms of study design, sample size, patient characteristics, AF type distribution, ablation strategies, biomarker assays, and follow-up duration, which may have influenced the strength or consistency of reported associations. Second, most studies were conducted in single-center cohorts, with a substantial proportion from China, thereby potentially restricting applicability of the results to more diverse patient populations. Third, not all biomarkers were evaluated in large cohorts; several emerging biomarkers were evaluated in studies with small sample sizes, increasing the risk of type I error and publication bias. Fourth, due to significant heterogeneity across included studies, a pooled quantitative meta-analysis was not conducted, which limits the ability to derive aggregated effect estimates. Fifth, the restriction to studies published within last 5 years and those with freely accessible full text may have excluded relevant earlier or subscription-based studies, introducing potential selection bias. Sixth, certainty of evidence using GRADE methodology was not formally assessed due to substantial heterogeneity and the qualitative nature of the synthesis. Seventh, given the geographic concentration of studies, particularly from single centers or specific regions, overlap of patient populations across publications cannot be entirely excluded. Although efforts were made to identify duplicate cohorts, incomplete reporting of enrollment periods limits certainty. Potential overlap may influence perceived consistency of associations. Finally, although this review only included studies with at least 1 year of follow-up, the absence of standardized long-term follow-up and the exclusion of non-English language may have led to omission of relevant data.

## Conclusion

This systematic review identifies multiple conventional and emerging biomarkers associated with AF recurrence after catheter ablation. While causality cannot be inferred, markers such as BMP-10, CA-125, Hcy, TyG index, NT-proBNP, and MR-proANP demonstrate stronger consistent associations and may hold potential for improving risk stratification. Future multicenter studies should evaluate whether incorporation of selected biomarkers into clinical risk models can meaningfully improve patient selection, post-ablation monitoring strategies, and personalized management approaches.

## Data Availability

The authors declare that data supporting the findings of this study are available within the article.
